# Case report: Clearance of longstanding, immune-deficiency-associated, vaccine-derived polio virus infection following remdesivir therapy for chronic SARS-CoV-2 infection

**DOI:** 10.3389/fimmu.2023.1135834

**Published:** 2023-03-03

**Authors:** William Hywel Bermingham, Benjamin Canning, Thomas Wilton, Michael Kidd, Dimitra Klapsa, Manasi Majumdar, Kavitha Sooriyakumar, Javier Martin, Aarnoud P. Huissoon

**Affiliations:** ^1^ Department of Immunology, University Hospitals Birmingham NHS Foundation Trust, Birmingham, United Kingdom; ^2^ Department of Virology, Nottingham University Hospitals NHS Trust, Nottingham, United Kingdom; ^3^ Division of Vaccines, National Institute for Biological Standards and Control, Medicines and Healthcare Products Regulatory Agency, Potters Bar, United Kingdom; ^4^ Public Health Laboratory, UK Health Security Agency, Birmingham, United Kingdom

**Keywords:** polio, vaccine derived poliovirus, immune deficiency associated vaccine derived poliovirus (iVDPV), remdesivir, CVID - common variable immunodeficiency disorders, primary immune deficiencies (PID)

## Abstract

The global polio eradication campaign has had remarkable success in reducing wild-type poliovirus infection, largely built upon the live attenuated Sabin oral poliovirus vaccine. Whilst rare, vaccine poliovirus strains may cause infection and subsequently revert to a neurovirulent type, termed vaccine-derived poliovirus (VDPV). Persistent, vaccine derived infection may occur in an immunocompromised host (iVDPV), where it is a recognised complication following receipt of the Sabin vaccine. This has significant implications for the global polio eradication campaign and there is currently no agreed global strategy to manage such patients.Here we describe a case of a 50-year-old man with common variable immune deficiency, persistently infected with a neurovirulent vaccine-derived type 2 poliovirus following vaccination in childhood. iVDPV infection had proven resistant to multiple prior attempts at treatment with human breast milk, ribavirin and oral administration of a normal human pooled immunoglobulin product. His iVDPV infection subsequently resolved after 12 days treatment with remdesivir, an adenosine analogue prodrug that is an inhibitor of viral RNA-dependent RNA polymerase, administered as treatment for a prolonged, moderate severe acute respiratory syndrome coronavirus 2 (SARS-CoV-2) infection. iVDPV from the patient, isolated prior to treatment, was subsequently demonstrated to be sensitive to remdesivir in vitro. Based on the observations made in this case, and the mechanistic rationale for use with iVDPV, there is strong justification for further clinical studies of remdesivir treatment as a potentially curative intervention in patients with iVDPV infection.

## Case history

The global polio eradication campaign has had remarkable success in reducing wildtype polio (WTP) virus infections (1). This success has largely been built on the use of the live attenuated Sabin oral polio virus (OPV), which has been in widespread use since 1962, although this has been superseded by the use of an inactivated virus in much of the world. The OPV has resulted in a dramatic reduction in wild-type polio cases. Although it is rare, the live OPV vaccine is associated with vaccine-associated paralytic poliomyelitis, particularly in the immunocompromised. The vaccine strains can revert to a neurovirulent type following replication in humans. With wild-type infections now suppressed throughout much of the world, vaccine-derived poliovirus (VDPV) infections present a significant challenge to the WHO global polio eradication initiative (2). VDPV has significant sequence divergence compared to the parent strain (>1% sequence divergence (PV1 and PV3) or >0.6% (PV2) in the VP1 gene). The detection of these divergent strains impliesmanymonths of ongoing viral replication. This may arise either in the context of prolonged spread in settings of low population immunity or with persistent infection in an immunocompromised individual. The VDPV detected due to ongoing community spread is termed circulating VDPV (cVDPV), whilst that from persistent infection in immunodeficient hosts is termed immunodeficiency-associated VDPD (iVDPV) (2). Patients with iVDPV are predominantly reported in those with severe combined immune deficiency or hypogammaglobulinaemia (3). These cases are rare, but significant, with 149 cases reported between 1961 and 2019 (3). iVDPV infections are, by definition, persistent for >6 months, may demonstrate highly virulent and antigenically modified virus, and can cause paralytic illness (3, 4). It is recognised that such patients have the potential to causewider outbreaks of iVDPV, but, to date, no data exist to quantify this risk, and there is no agreed global strategy to manage these patients (1, 4). Here, we report, to our knowledge, the first case of resolution of longstanding iVDPV infection in an individual with common variable immune deficiency (CVID, a primary antibody deficiency) following a prolonged, moderate severe acute respiratory syndrome coronavirus 2 (SARS-CoV-2) infection where the patient received a total of 12 days of treatment with remdesivir.

**Figure 1:**
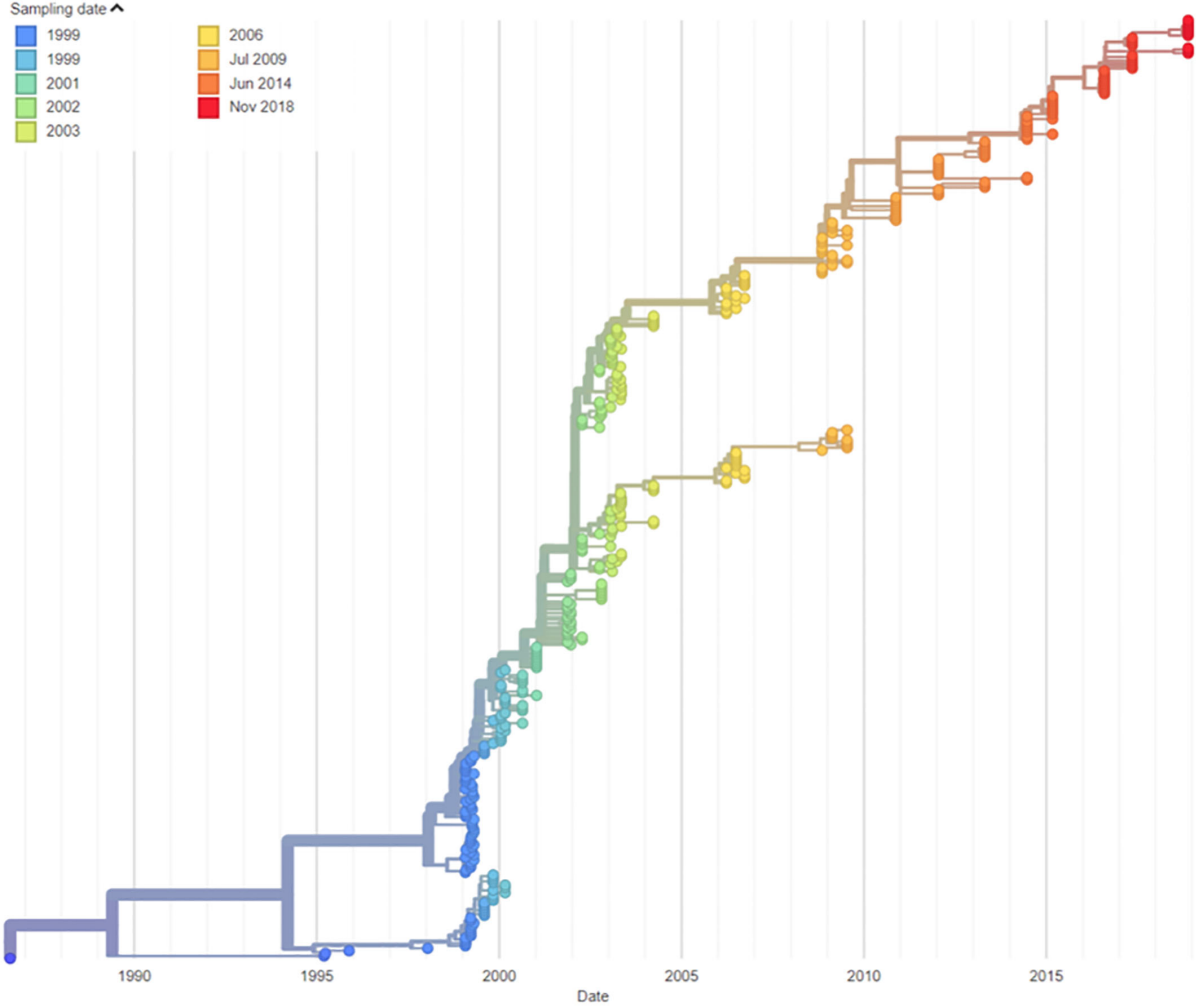
Patient iVDPV sequence evolution and molecular clock analysis. .

The patient is a 50-year-old man who was diagnosed with CVID at the age of 15, having presented with a history of recurrent respiratory infections. He commenced continuous immunoglobulin replacement therapy following diagnosis. This was initially *via* intramuscular route, followed by intravenous immunoglobulin from age 19 and subcutaneous immunoglobulin from age 41. He remained under regular specialist immunology care throughout this time. He had received a full course of UK childhood immunisations, including oral polio vaccine at age 5, 7, and 12 months, plus additional booster doses at 7 and 14 years of age. iVDPV infection was first identified in 1995 (age 23 years) when type 2 poliovirus was cultured from the stool. Type 2 polioviruses are classified as a hazard group 3 pathogen by the UK Advisory Committee on Dangerous Pathogens (5). He reported a longstanding bowel habit of one to two stools per day, which were occasionally loose in nature (estimated Bristol stool classification 4–6). He reported no neurological symptoms relevant to his poliovirus carriage throughout the period where this was identified. Viral isolates from this patient were demonstrated to be highly neurovirulent, with further details on their characterisation previously reported (4). Molecular clock nucleotide sequence analysis confirmed the date of vaccine administration as compatible with the patient’s last known OPV vaccination on 4 August 1986 (**Figure 1**).

**Figure 2:**
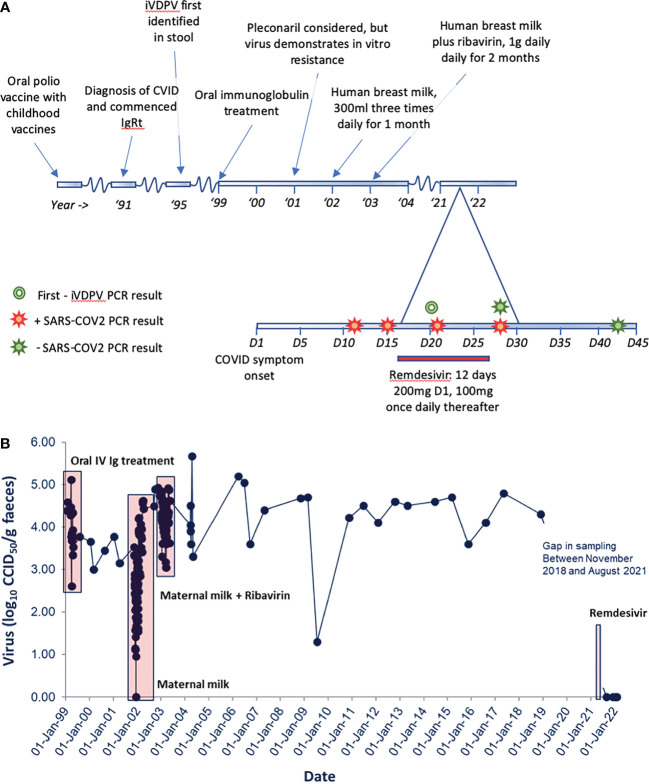
**(A)** A graphical representation of clinical timeline and relevant microbiological results. **(B)** iVDPV stool viral load on serial samples tested at the National Institute for Biological Standards and Control (NIBSC). Unsuccessful trials of treatment for iVDPV are displayed from 1999 to 2003. Persistent iVDPV carriage is then demonstrated to last positive sample, November 2018. Initial negative stool samples following remdesivir therapy are shown. Patient stool samples remain negative to August 2022.

From 1999 to 2003, a number of attempts were made to clear iVDPV infection, including regular oral administration of human breast milk (with and without the addition of ribavirin) and oral administration of a normal human pooled immunoglobulin product). Pleconaril treatment was considered but not utilized due to *in vitro* resistance of the virus to this agent. These treatment strategies resulted in transient reductions in stool virus load but failed to clear the iVDPV infection (**Figure 2B**). Further details of these treatments are available in a previously published work (6). The patient remained under regular routine iVDPV surveillance and recorded positive stool cultures by virus cell culture isolation and PCR assays in all stools (n = 27) tested between 1 May 2003 and 27 November 2018 with high viral loads, typically between log_10_ 4.0 and 5.0 infectious units per gram of stool. This means that he had been excreting type 2 poliovirus for more than 32 years by that time. No further stools were tested after this due to difficulties in sending samples in the context of stringent WHO biosafety containment recommendations for handling poliovirus infectious materials.

In August 2021, the patient developed symptoms consistent with severe acute respiratory syndrome coronavirus 2 (SARS-CoV-2) infection with pyrexia, malaise, rigours, and cough (day 1). He subsequently confirmed infection with positive nucleic acid amplification test (NAAT) on day 12. He had taken a “rescue” course of doxycycline as per his normal specialist management plan for suspected exacerbation of bronchiectasis without appreciable benefit. Symptoms were persistent with headache, anosmia, aguesia, malaise, cough, and recurrent pyrexia on day 15 of infection, prompting admission to hospital for treatment with intravenous remdesivir from day 16. On admission, he was febrile with a NEWS2 score (7) of 2 (pyrexia/tachycardia); C-reactive protein (CRP) was raised at 59 mg/L (0–5 mg/L). He received 200 mg IV remdesivir on day 1, followed by daily administration of 100 mg IV remdesivir. This was based on local multidisciplinary specialist discussion (clinical immunology/virology/infectious diseases specialists) and our and others’ experience for the treatment of SARS-CoV-2 in patients with hypogammaglobulinemia. No adverse events relating to treatment were reported. SARS-CoV-2-specific neutralising monoclonal antibodies were not available for this patient’s treatment (8).

Following initiation of remdesivir therapy, the patient noted improvement in his symptoms, and pyrexia resolved after 3 days of treatment. Remdesivir therapy was continued for a total course of 12 days, as SARS-CoV-2 RNA was consistently detected by reverse transcriptase quantitative polymerase chain reaction (RT-qPCR) testing on nose/throat swabs. Whilst remaining detectable, RT-qPCR cycle threshold increased, suggesting a reduction in SARS-CoV-2 viral load. He was discharged on day 27, feeling much improved, apyrexial, and describing “75%” recovery of taste. He subsequently reported ongoing gradual symptomatic recovery, his inflammatory markers improved, and SARS-CoV-2 was not detected by RT-qPCR on day 43. The patient made a detectable specific T-cell response to SARS-CoV-2 spike and nucleocapsid proteins by a validated in-house interferon gamma release assay. Anti-SARS-CoV-2 spike IgG was also detectable both during admission and subsequently with increasing titres. However, the relative contribution of his normal human immunoglobulin replacement product (Hizentra© 20% subcutaneous normal human immunoglobulin) to this is uncertain. A stool sample from day 20 (fifth day of remdesivir therapy) was negative for poliovirus, and subsequent samples collected in December 2021, and monthly up to and including August 2022, remained negative for poliovirus in virus isolation and PCR assays. The patient reported no significant change in his longstanding bowel habit prior to, during, or following remdesivir therapy. He reported no significant change in his normal bowel habit between iVDPV-positive and iVDPV-negative samples. Clinical course and stool sampling for iVDPV are summarised in **Figure 2**.

Given the possibility of remdesivir having caused this unprecedented interruption of VDPV excretion in the patient, the sensitivity to remdesivir of his last stool poliovirus isolate from 27 November 2018 was tested in cell culture assays (9). Remdesivir was tested alongside other known antiviral products used for compassionate treatment including pocapavir, the only available antiviral known to be effective against some poliovirus isolates, and other products with known antiviral activity against other enteroviral infections (10). The virus was found to be sensitive to remdesivir and pocapavir, demonstrating inhibition of 100 infectious doses of virus at 1 and 0.5 μM concentrations, respectively. Antivirals ribavirin, favipiravir, and fluoxetine hydrochloride did not inhibit virus growth at concentrations ≤50 µM used in the assays.

## Discussion

There is a need for effective antiviral treatments to manage the threat of iVDPV infections to the global polio eradication strategy. These would directly inform a coordinated, global response to these patients, which is currently lacking. Whilst no VDPV outbreaks have been directly linked to iVDPV cases, affected individuals have been reported to experience paralytic illness and also shed highly neurovirulent viral isolates. A recent detection of VDPV type 2 poliovirus in London sewage demonstrates that this is a demonstrable risk to populations (11).

iVDPV cases may persist for many years, with our patient being (to the authors’ knowledge) the longest reported case of continuous iVDPV shedding. Prior to the events described here, this patient’s infection had been resistant to treatment strategies attempted. This is consistent with other case reports. Where cases of iVDPV have been demonstrated to resolve, one occurred following haematopoietic stem cell transplant (12). Another intriguing case of a patient with SCID resolved spontaneously following a norovirus infection. However, this case had only been identified to carry iVDPV for 2 years prior to reported resolution (12).

Remdesivir is a monophosphoramidate adenosine analogue prodrug that has seen widespread use during the coronavirus disease 2019 (COVID-19) pandemic as an inhibitor of SARS-CoV-2 RNA-dependent RNA polymerase (RdRp). It has antiviral activity against a broad range of RNA viruses *in vitro*. It has been demonstrated to inhibit the replication of enterovirus 71 (EV71) following cell entry and to inhibit viral complementary RNA (cRNA) production in culture. It also inhibits coxsackievirus B3 (CVB3) and enterovirus 70 (EV70) in culture. Whilst it has not been explicitly demonstrated to inhibit poliovirus, the conserved nature of viral RdRps suggests that it likely has activity against the poliovirus RdRp, too (13).

This case report presents a number of limitations. Retrospectively, it is not possible to determine the relative contribution of the patient’s prolonged SARS-CoV-2 infection, associated pro-inflammatory state, and remdesivir therapy in the elimination of iVDPV carriage in this patient. It is noteworthy that cross-reactivity has been demonstrated in poliovirus-vaccinated sera to SARS-CoV-2 RdRp antigen, although the clinical relevance of this is uncertain (14). Furthermore, as stool sampling prior to presentation with SARS-CoV-2 infection was intermittent, it is possible that iVDPV carriage could have spontaneously remitted. However, this seems extremely unlikely in light of his consistent and longstanding carriage for over 32 years and prior resistance to targeted therapy. Furthermore, his last VDPV isolate was shown to demonstrate sensitivity to remdesivir therapy in cell culture laboratory assays.

Based on the observations made in this case, and the mechanistic rationale for use with iVDPV, there is strong justification for further clinical studies of remdesivir treatment as a potentially curative intervention in patients with iVDPV. This has the potential to inform iVDPV management strategy as an important cornerstone of the future WHO global polio eradication initiative.

## Patient perspective

“I was not told until 1998 that I was shedding polio virus. Subsequently a number of options were tried including frozen human milk from donors *via* Birmingham Women’s Hospital baby unit and drug trials. The milk seemed like it was working, and we persevered despite tasting terrible. Unfortunately, after a break (due to supply issues), the effects dropped off. Other potential drugs were proposed by various immunologists and some tested by NIBSC, but nothing was suitable. Unfortunately, after keeping healthy through the first 16 months of the pandemic, I caught COVID-19 along with the rest of our household at the beginning of August 2021. I continued to have a very high temperature after 2 weeks, and after contacting Heartlands, the immunologists recommended that I should come into hospital for observation and to receive the experimental treatment of the anti-viral Remdesivir. This started to have an effect on the COVID symptoms; temperature and hallucinations receded, and I continued on it for about 2 weeks.

While on the drug in the hospital, I researched online and was interested to read about it affecting enterovirus and whether it would also have any effect on polio, which the immunologists also excitedly brought to my attention. I contacted NIBSC asking if they could arrange testing. Over the last few years, as regulations changed, testing had become extreme, requiring an expensive courier direct to the testing lab so had become infrequent, and of course, no change had been detected for years. NIBSC contacted HSE, and we were able to arrange my samples to be collected and transported for testing, which I coordinated with them and HSE. A few weeks later, in September 2021, I learned that those samples were all negative for polio, which was amazing to hear. They continued to be negative as I tested monthly until over a year had passed. I think it is almost certain that the polio was still present when I caught COVID. I can only assume remdesivir was what cleared it, although I also had a very high temperature and ate very little, so I had little gut movement.

I will be very interested to hear of future studies of this drug against other people with this condition and ultimately the help that it may give in the polio eradication programme.”

## Data availability statement

The raw data supporting the conclusions of this article will be made available by the authors, without undue reservation.

## Ethics statement

Written informed consent was obtained from the individual(s) for the publication of any potentially identifiable images or data included in this article.

## Author contributions

WB: Manuscript authorship and review. Collation of data and figures. Clinical case management. BC: Manuscript authorship and review. Collation of data. Clinical case management. TW: Manuscript authorship and review. Collation of data and figures. Analysis of samples MK: Manuscript critical review. Clinical case management. DK: Collation of data and figures. Analysis of samples MM: Collation of data and figures. Analysis of samples KS: Review of manuscript. Clinical case management. JM: Senior Authorship. Collation of data and figures. Analysis/interpretation of samples AH: Senior Authorship. Collation of data and figures. Clinical case management. All authors contributed to the article and approved the submitted version.
